# Prematurity alters the progenitor cell program of the upper respiratory tract of neonates

**DOI:** 10.1038/s41598-021-90093-x

**Published:** 2021-05-24

**Authors:** Jessica E. Shui, Wei Wang, Helu Liu, Anna Stepanova, Grace Liao, Jun Qian, Xingbin Ai, Vadim Ten, Jining Lu, Wellington V. Cardoso

**Affiliations:** 1 Columbia Center for Human Development, Pulmonary Allergy & Critical Care Medicine, Department of Medicine, Columbia University Irving Medical Center, 650 West 168th Street, BB 8-812, New York, NY 10032 USA; 2 Division of Neonatology, Department of Pediatrics, Columbia University Irving Medical Center, New York, NY USA; 3 Division of Neonatology and Newborn Medicine, Department of Pediatrics, Massachusetts General Hospital, Harvard Medical School, Boston, MA USA; 4 Division of Lung Diseases, NHLBI, NIH, Bethesda, MD USA

**Keywords:** Multipotent stem cells, Paediatric research, Translational research

## Abstract

The impact of prematurity on human development and neonatal diseases, such as bronchopulmonary dysplasia, has been widely reported. However, little is known about the effects of prematurity on the programs of stem cell self-renewal and differentiation of the upper respiratory epithelium, which is key for adaptation to neonatal life. We developed a minimally invasive methodology for isolation of neonatal basal cells from nasopharyngeal (NP) aspirates and performed functional analysis in organotypic cultures to address this issue. We show that preterm NP progenitors have a markedly distinct molecular signature of abnormal proliferation and mitochondria quality control compared to term progenitors. Preterm progenitors had lower oxygen consumption at baseline and were unable to ramp up consumption to the levels of term cells when challenged. Although they formed a mucociliary epithelium, ciliary function tended to decline in premature cells as they differentiated, compared to term cells. Together, these differences suggested increased sensitivity of preterm progenitors to environmental stressors under non-homeostatic conditions.

## Introduction

The respiratory tract epithelium represents the large interface of the body with the outside environment and primarily functions to conduct air and to facilitate its transport through the alveolar-capillary barrier for gas exchange. The conducting airways are crucial for cleaning the air from particulates and biological agents and are responsible for metabolizing inhaled toxic compounds and environmental pollutants. These functions are exerted by a variety of differentiated airway cell types, including distinct secretory (goblet, club cells), multiciliated and other minor populations (neuroendocrine, ionocytes) regionally balanced from the nasal passages to the terminal bronchioles^1–4^.

The various populations of differentiated luminal airway cells arise and are maintained by a pool of undifferentiated stem/progenitor cells (stem cell compartment) largely comprised of basal cells, multipotent progenitors basally located along the basement membrane^2^. By turning on specific programs of cell fate, these multipotent progenitor cells acquire molecular and morphological features to generate the various luminal cell types. Studies in mouse genetic models show that basal cells form during prenatal development and together with undifferentiated embryonic precursors, contribute to the pool of differentiated luminal cells^5^. The transition from prenatal to postnatal life imposes major challenges to the epithelium that must respond to the markedly different hyperoxic environment triggered by the first breath, as well as have an innate immune mechanism able to handle exposure to biological agents not present during intrauterine life. Given that these depend on differentiation/maturation events that are completed largely by the end of the third trimester, premature birth can be associated with respiratory diseases of the neonate with increased morbidity and mortality.

While the impact of prematurity on the differentiated luminal cells of the airway epithelium has been reported in diseases such as bronchopulmonary dysplasia, little is known about how it affects the stem cell compartment of the airways^6–12^. Are the intrinsic programs of self-renewal and cell fate of basal cells affected by premature birth in infants? If so, how and to which extent this impairs the ability to form specific cell types? If exposed to the same physiologic normal conditions can premature and mature stem cell develop similarly? These compelling questions are of high significance but difficult to systematically address. Assessment of the intrinsic properties of basal stem/progenitor cells from neonates would require invasive procedures, such as intubation, trachea/bronchial brushings or even biopsy. Clinically, these opportunities would not occur in healthy term or preterm newborns in neonatal intensive care unit (NICU) settings.

Here we have developed a minimally invasive methodology for systematic isolation and functional analysis of the stem cell properties of human basal cells from nasopharyngeal aspirates. We show that these cells can be expanded and passaged without losing their original identity or behavior. We used this system as a platform to investigate how prematurity influences the stem cell behavior and the differentiation capacity of airway basal cells in preterm compared to term neonates. RNAseq showed remarkably distinct molecular signatures between basal cells from these groups, with preterm cells showing features of mitochondrial dysfunction and functional evidence of decreased oxygen consumption. Prematurity did not prevent epithelial differentiation but still maintained altered gene signature and showed a decline in ciliary beating surface area, suggesting that the epithelium may be more susceptible to environmental challenges and injuries.

## Results

### Nasopharyngeal aspirates from newborns are a source of epithelial progenitors for studying the stem cell program of the upper respiratory tract

Nasopharyngeal (NP) aspiration is a routine procedure in newborn care widely used to help clear the airways from residual amniotic fluid and secretions^13^. This minimally invasive procedure is performed within the first minutes of neonatal life and consists of suctioning the remaining fluid in the upper airspaces (nasal, oropharynx) using a soft canula. NP aspirates are routinely discarded and known to be comprised of a loosely defined mixture of airway epithelial, inflammatory and amniotic cells in abundant mucous secretion. We reasoned that these aspirates included a stem cell population that, even if small, could provide an opportunity to investigate the program of self-renewal and differentiation of the airway epithelium of these neonates.

**Figure 1:**
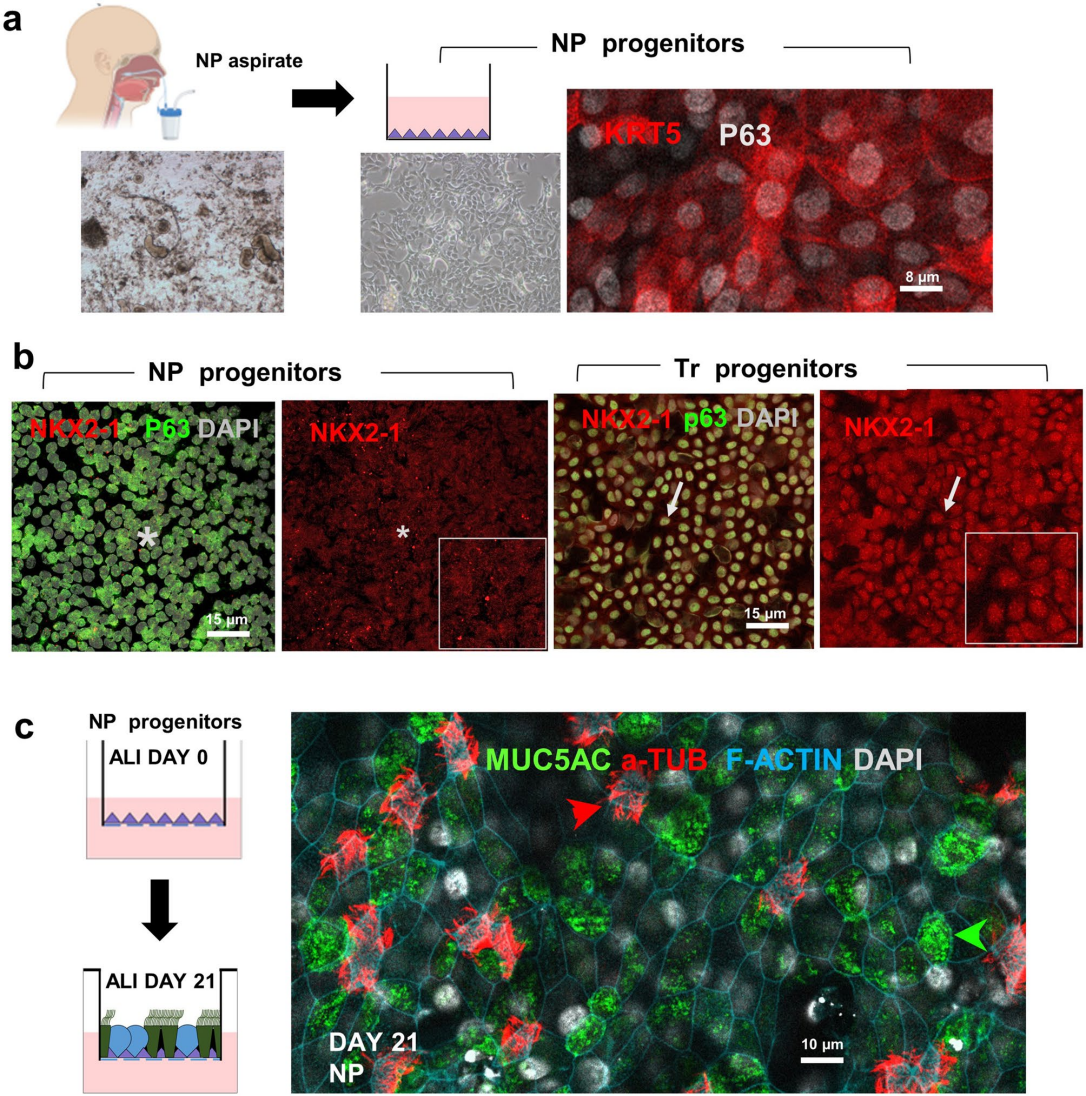
Epithelial progenitors from nasopharyngeal (NP) aspirates of newborns expand and differentiate in organotypic cultures. (**a**) NP aspirate collection at birth (left: mixed cells, debris). Culture and selective expansion to confluency of NP epithelial progenitors shown to be basal cells (phase contrast image and immunostaining with P63 and KRT5). (**b**) NP progenitors are NKX2-1 negative basal cells of the upper respiratory tract (left: confluent plate p63+) in contrast to tracheal-derived progenitors cultured under same conditions, which are NKX2-1+ p63+ basal cells (right). (**c**) Neonatal NP-derived basal cells undergo differentiation if cultured in air–liquid-interface (ALI), as shown by immunostaining for multiciliated (acetylated a-tubulin) and goblet (MUC5AC) cells; F-Actin and DAPI depict cell boundaries and nuclei, respectively. Insets in (**b**) depict positive (arrow) or negative (*) signals. Bars (**a**–**c**) represent 8, 15, and 10 µm, respectively.

To investigate this issue, we collected NP aspirates from healthy term newborns (37–40 weeks gestation) and seeded approximately 3.5 × 10^4^ cells from each sample onto collagen-coated Transwell inserts. Cells were cultured in BEGM medium under submerged conditions until confluency (7–10 days), as similarly reported for primary cultures of tracheobronchial progenitor cells^14^. Immunofluorescence (IF) and confocal analysis of confluent NP cells showed the extensive P63+, KRT5+ labeling characteristic of basal cells (Fig. 1a). Interestingly, IF for NKX2-1, which marks the tracheobronchial lineage, showed no signals in NP-derived P63+ cells, in contrast to the tracheal P63+ basal cells from adult organ donors cultured under similar conditions (Fig. 1b). Thus, NP and tracheal basal cells are multipotent progenitors of distinct lineages and developmental origins^15^. The lack of NKX2-1 positive cells in the NP confluent colonies indicated that the aspirates did not contain basal cells from lower regions of the respiratory tract using our methodology for sample collection and cell expansion. However, NKX2-1 negative/p63+ progenitors in these aspirates could be from different origins, including the oral or esophageal epithelium.

Thus, we examined the developmental potential of this NP progenitor cell pool by culturing them under air–liquid-interface (ALI) using conditions previously established for primary human tracheobronchial epithelial cells^14,16^. Analysis of these cultures over the following three weeks post ALI conditions showed a pattern of differentiation similar to that reported for tracheobronchial cells with abundant secretory (MUC5AC+) and multiciliated (a-TUB+) cells (Fig. 1c). This differentiation profile indicated that NKX2-1 negative/P63+ NP basal cells represented the progenitors of the airway epithelium of the upper respiratory tract (airways above larynx), a region of crucial first line innate immune defense mechanism. Thus, organotypic cultures derived from NP aspirates could be used to investigate the progenitor cell behavior and differentiation potential of donor-derived cells from newborns.

### Transcriptome analysis of NP-derived neonatal progenitors identifies signatures common to adult basal cells of the upper respiratory tract

In neonatal settings prematurity represents the single most important factor influencing mortality and long-term morbidity, including lung conditions associated with aberrant developmental programs^10,17–19^. The understanding of these programs has been hindered by the paucity of systems to properly assess the intrinsic properties of the epithelial barrier and their response to the extra-uterine environment. We reasoned that the analysis of the NP aspirates (above) could provide an opportunity to study the impact of prematurity in the epithelial barrier in neonates. Thus, we collected NP aspirates from preterm neonates (23 0/7–30 6/7 weeks gestational age) and healthy term neonates (37 0/7–40 6/7 weeks gestational age) within the first minutes of life, as part of our delivery room resuscitation procedures at CUIMC, and cultured as described above.

**Figure 2:**
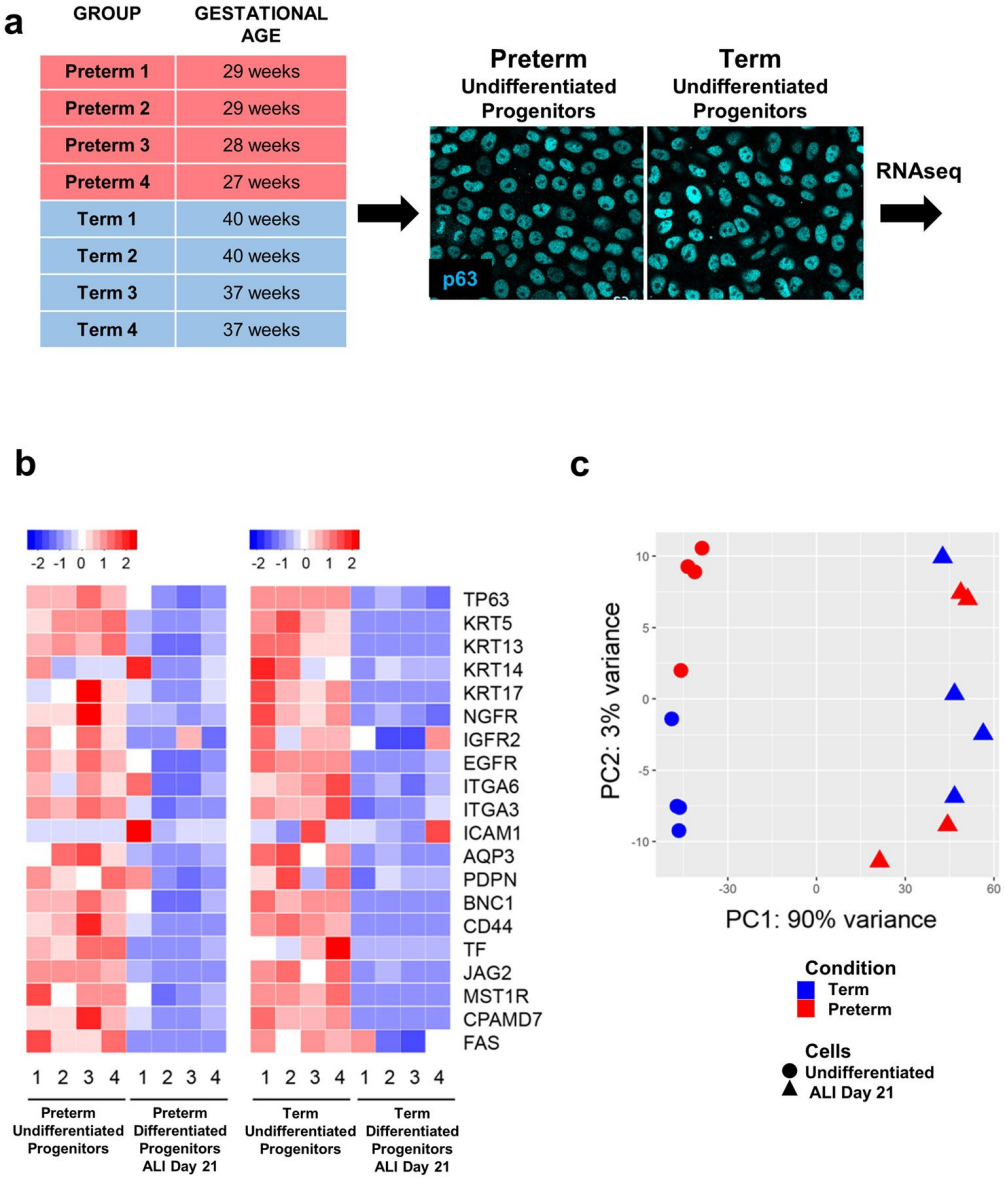
Transcriptome analysis confirms identity of undifferentiated NP-derived progenitors as basal cells. (**a**) NP basal cells from newborn subjects (preterm n = 4 and term n = 4) for bulk RNAseq analysis (left); P63 immunostaining depicting similar signal intensity of confluent preterm and term undifferentiated progenitor basal cells. (**b**) Heat map showing expression of known markers of human airway basal cells in the RNAseq dataset from preterm and term cultured NP progenitors before differentiation and after 21 days of differentiation in ALI conditions. (**c**) Principal Component Analysis (PCA) of preterm and term cultures of NP progenitors prior to and after differentiation (ALI day 21) showing clear separation between samples in undifferentiated but not in differentiated cultures.

First, we asked whether we could identify changes in the transcriptomics of the stem cell compartment of the NP epithelium, making preterm and term progenitors intrinsically different when expanded under similar culture conditions. Preterm and term cells (4 subjects per group) reached confluency within 7–10 days and showed the cobblestone morphology with strong nuclear P63 labeling typical of basal cells (Fig. 2a). Three NP confluent plates from each subject were harvested for RNA isolation while the remaining set of cultures was allowed to differentiate under ALI conditions until day 21 (described in a subsequent section). RNA-seq libraries from NP progenitors were sequenced and analyzed for differentially expressed genes between preterm and term groups (Illumina, NovaSeq 6000, JP Sulzberger Columbia Genome Center, NY).

Our organotypic cultures (Fig. 1) provided evidence that the p63+ basal cells isolated from the nasopharyngeal aspirates functioned as multipotent progenitors. However, it was unclear the extent to which they acquired the gene repertoire of mature basal cells since they originated from newborn subjects.

Thus, we assembled a panel of markers of mature basal cells previously identified by transcriptome analysis of human nasal, tracheobronchial and similarly cultured primary cells^3,16,20–22^. Then, we looked for expression of these genes in our NP progenitor RNAseq dataset. Analysis of transcript abundance showed that, in spite of their neonatal origin, the NP progenitors expressed all twenty canonical basal cell markers (TP63, KRT5, KRT13, KRT14, KRT17, NGFR, IGFR2, EGFR, ITGA6, ITGA3, ICAM1, AQP3, PDPN, BCN1, LHR, TF, JAG2, MST1R, CPAMD7, FAS). Notably, expression of these genes decreased dramatically as they differentiated in both preterm and term cells, supporting their enrichment at the progenitor cell stage (Fig. 2b, c). Together the observations confirmed the basal cell identity of these progenitors and suggested that prematurity does not prevent the program of acquisition of the basal cell phenotype as they expanded in culture. Our RNA-seq analysis also confirmed that the NP-derived basal cells are NKX2-1 negative multipotent progenitors, do not express NKX2-1 mRNA and thus do not originate from the lung primordial cells.

### Prematurity is associated with a gene signature of susceptibility to a maladaptive cellular response in NP progenitors

Next, we conducted a Principal Component Analysis (PCA) of preterm and term cultures to have an idea of the overall effect of prematurity on the transcriptome of NP progenitors prior to and after differentiation (DEseq2 package). Intriguingly, while PCA showed no clear separation between the preterm and term samples in differentiated cultures (ALI day 21), a distinct pattern of distribution was identified in NP progenitors prior to differentiation (Fig. 2c). Strikingly, further analysis showed 2737 differentially expressed genes in preterm and term NP basal cells (p-adjusted < 0.05). Thus, their identity as basal cells was preserved, but prematurity made a major impact on the transcriptome with possible functional consequences in their ability to self-renew or differentiate.

**Figure 3:**
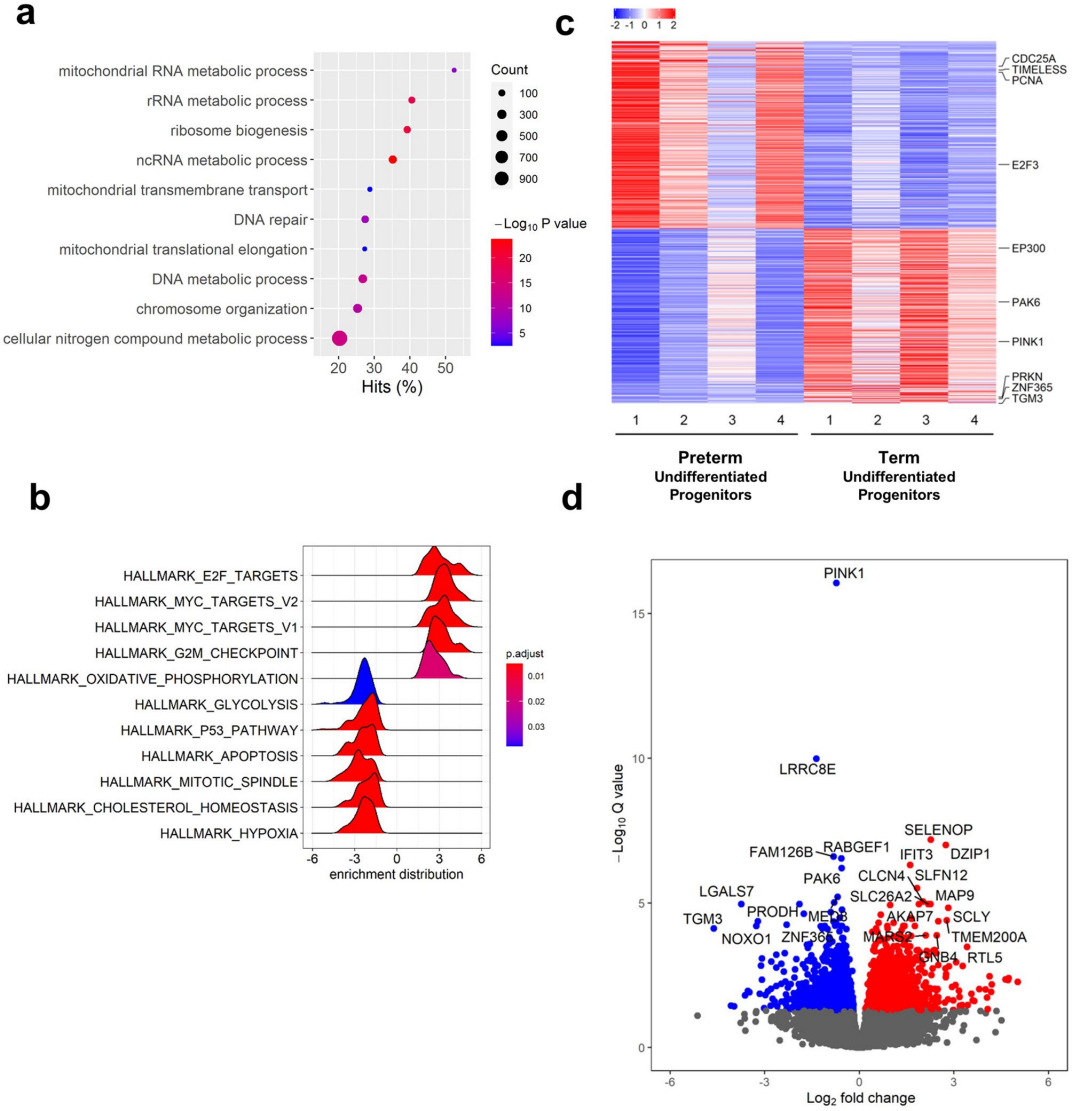
Prematurity is associated with a distinctive gene expression signature in NP-derived basal cells. (**a**) Dot plots of top 10 enriched GO terms based on overrepresented p-value generated by *goseq* in undifferentiated confluent cultures of preterm vs term NP basal cells. Dot size represents the number of genes from a particular GO term differentially expressed (count), color depicts the p-value, and hit (%) refers to the percentage of differentially expressed genes in a GO term. (**b**) Ridge plots of all significantly enriched pathways by GSEA analysis (p-adjusted value < 0.05) in these cultures. (**c**) Heat map depicting gene expression signatures of each preterm and term samples (confluent basal cells before differentiation). (**d**) Volcano plot highlighting the fold change and significance of the most differentially expressed genes Significantly regulated genes above and below twofold are shown in red and blue, respectively.

We performed hierarchical analysis and determined the gene categories enriched in each group to gain insights into potential relevant functional differences (Fig. 3). Gene ontology identified the most prevalent biological processes as rRNA metabolic process, RNA processing, and ribosome biogenesis. Hierarchical clustering and Gene Set Enrichment Analyses (GSEA) showed a large number of proliferation-related genes upregulated differentially in preterm cells, suggesting higher activity in cell expansion (Fig. 3a). Hallmark categories enriched included E2F targets, Myc targets V1, Myc targets V2, and G2-M checkpoint; other hallmark categories included interferon responses, DNA repair, oxidative phosphorylation, mTORC signaling and glycolysis (Fig. 3b). A closer look into the representative upregulated genes from these categories revealed Proliferating Cell Nuclear Antigen (PCNA) (p-adj = 0.001; 2.6-fold), Timeless Circadian Regulator (TIMELESS) (p-adj = 6.21 × 10^–5^; 2.7-fold), E2F Transcription Factor 3 (E2F3) (p-adj = 0.026, 1.57-fold), and Cell Division Cycle 25A (CDC25A) (p-adj = 9.45 × 10^–4^, 2.9-fold) (Fig. 3c). In addition, preterm cells showed proliferation-suppressor genes were significantly downregulated, including Transglutaminase 3 (TGM3) (p-adj = 7.60 × 10^–5^; 25 fold), and p21 Activated Kinase 6 (PAK6) (p-adj = 6.18 × 10^–7^, 1.47 fold), further supporting the idea of higher proliferation activity compared to term cells^23–25^ (Fig. 3c). Hallmark GSEA pathways downregulated in preterm included those associated with hypoxia, protein secretion, cholesterol metabolism and the p53 pathway (Fig. 3b).

We generated volcano plots from the preterm and term progenitor transcriptomes to highlight the most statistically significant genes differentially expressed at the highest fold changes. This revealed LGALS7 (p-adj = 10^–5^, 13-fold), PRODH (p-adj = 4.25 × 10^–5^, 9.3 fold), TGM3 (p-adj = 7.6 × 10^–5^, 24.5 fold), NOXO1 (p-adj = 6.21 × 10^–5^, 9.7 fold), and ZNF365 (p-adj = 5.60 × 10^–5^, fivefold) as the most downregulated genes in preterm progenitors (Fig. 3d). Unexpectedly, mining of curated public databases and literature search showed the majority of these genes were known P53-dependent targets^26,27^. GSEA confirmed the HALLMARK_P53_PATHWAY as one the most differentially expressed signatures with the highest number of target genes downregulated in preterm cells (Fig. 3b, Supplementary Fig. S1).

The tumor suppressor protein, P53, is a multifaceted transcription factor mostly known for its inhibitory effect in cell proliferation^28^. The downregulation of P53 targets and pathway associated genes in preterm cells correlated with the upregulation of proliferation-inducing (Ex. E2F targets, Supplementary Fig. S1) and downregulation of proliferation-suppressor genes in these cells. This was consistent with a preliminary finding of increased KI67 labeling in preterm compared to term basal cells (38.2 ± 1.4% vs 30 ± 1.6% five random fields, one culture per group). Paradoxically, P53 transcripts were more abundant in preterm compared to term cells (p = 0.001, 1.7-fold), suggesting that despite being expressed at higher levels, P53 was less activated in preterm than in term cells. Multiple studies show that P53 expression levels do not necessarily correlate with activity, which rather depend on P53 posttranslational modifications (acetylation phosphorylation), alternative splicing, among other factors^29^. In this regard, the histone acetylation EP300 (CBP/p300), an inducer of P53 activity was differentially downregulated in preterm (p = 0.027; 1.3-fold) (Supplementary Fig. S1). Subsequent studies beyond the scope of the present work will explore in depth these observations and mechanisms.

Environmental stressors are known to elicit reactive mechanisms in the stem cell pool that can lead to aberrant cell behavior programs^30^. We looked for a gene signature that could reveal the environmental stress impinged on preterm cells. The downregulation of genes associated with hypoxia suggested a potential role for oxygen as an environmental stressor. Oxidative phosphorylation is a key component of the normal metabolism but is also a source of deleterious reactive oxygen species (ROS) triggering cell damage and death. In stem cells, high ROS can alter cell metabolism, damaging lipids, proteins, DNA, and ultimately impair self-renewal^31^. Somewhat surprisingly, we could not find a gene signature consistent with oxidative stress in preterm cells. Gene markers, such as NEFL2 (NRF2), DDIT4, ATF3, HMOX, ENC1, SCO2, or those encoding antioxidant enzymes SODs, GPXs, PRDXs were not significantly altered^32^.

However, our RNAseq analysis showed that gene regulators of key protective cellular processes, such as mitochondria quality control, autophagic cell survival or apoptosis and determinants of cell cycle arrest/senescence, were abnormally expressed in preterm cells. Notably, preterm basal cells were significantly downregulated in the PTEN-induced kinase PINK1 (p-adj = 8.65 × 10^–17^, 1.7 fold) and the E3 Ubiquitin Protein Ligase PRKN (p-adj = 0.002, fourfold), two key mediators of autophagy responsible for mitochondria quality control and maintenance of cellular homeostasis and survival^33,34^ (Supplementary Fig. S1). Moreover, ZNF365 a direct p53 target required to prevent aberrant telomere damage and senesce was markedly downregulated in preterm progenitors (p-adj = 5.60 × 10^–5^, fivefold)^35^ (Fig. 3c,d). It is also noteworthy that genes involved in cholesterol homeostasis and heme metabolism were among the genes downregulated in these cells (Fig. 3b). GSEA revealed no significant enrichment in corticosteroid-responsive genes (p-adj = 0.343), suggesting that the preterm exposure to maternally-administrated steroid did not result in significant activation of steroid-responsive genes in the undifferentiated fetal NP progenitors.

Thus, prematurity appears to foster a program of self-renewal that favors expansion of progenitor cells less fit to adapt to environmental stressors, presumably with compromised ability to fully function.

### Decreased mitochondrial activity in preterm compared to term NP progenitor cells

**Figure 4:**
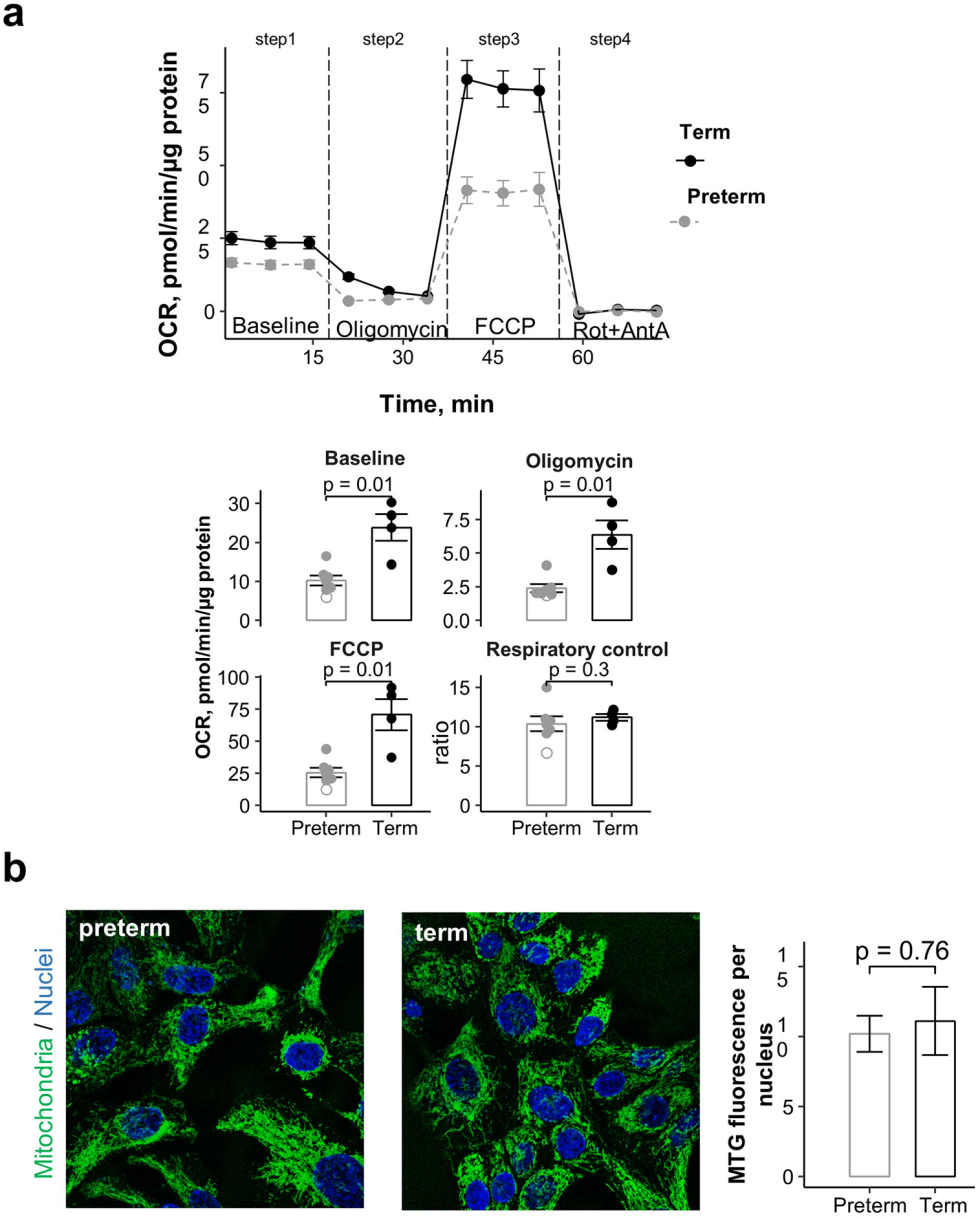
Diminished mitochondrial activity in preterm NP-derived basal cells. (**a**) Seahorse analysis of cellular oxygen consumption rate (OCR) over time in cultured NP-derived basal cells from preterm and term subjects; effects of selected compounds on different steps of the mitochondrial respiratory chain. OCR quantification at Baseline, Oligomycin-treatment, FCCP treatment and the cellular respiratory control ratio calculated as a ratio of the FCCP rate to Oligomycin rate. Each point represents an individual infant (n = 4 for term infants and n = 7 for premature infants). Open circle marks the premature infant with the youngest gestational age in the study (25 weeks gestational age). Bar graphs are mean ± SEM; p-values of the Mann–Whitney *U* test are shown. (**b**) MitoTracker Green (MTG) FM fluorescence staining in preterm and term confluent basal cells in culture (DAPI, blue). Graphs: bars are mean (± SEM) of MTG per nucleus in preterm and term cells; p-value (depicted) Student *t* test.

Our transcriptome analysis of preterm and term NP progenitors revealed significant differences in expression of genes associated with mitochondrial integrity and function. To investigate whether these changes reflected in differences in the cellular bioenergetics we analyzed the cellular mitochondrial respiration profile of these two populations (Seahorse XFe24 Agilent) (Fig. 4, Supplementary Fig. S2a). The cellular oxygen consumption rate (OCR, pmol of O_2_/min/μg protein) can be used to characterize the cellular bioenergetics at different states of mitochondrial respiration (baseline, resting, maximal) and define the capacity of mitochondrial respiratory chain to phosphorylate ADP to ATP.

NP progenitors from preterm (n = 5) and term (n = 4) newborns were expanded to confluency in XF cell culture microplates and incubated with Agilent Seahorse XF Base Medium for one hour prior to the assay. OCR was measured in three independent experiments (5–6 technical replicates per plate), under unchallenged baseline conditions (step 1), after adding the ATPase inhibitor oligomycin to determine the resting/residual OCR once proton-motive force is no longer required for ATP synthesis (step 2), then after adding the mitochondrial uncoupler FCCP to dissipate the proton-motive force and stimulate high oxygen consumption (step 3), and lastly, inhibiting respiratory chain complex I and III (rotenone and antimycin A, Rot + AntA), to abolish mitochondrial oxygen consumption and dissect out mitochondria non-specific cellular OCR (step 4). To control for mitochondrial mass, the mitochondria content of preterm and term NP progenitors was assessed using MitoTracker Green FM (Invitrogen).

We found significantly decreased baseline OCR in preterm compared to term cells, suggesting that in an aerobic environment and unchallenged homeostatic conditions, premature NP progenitors are metabolically less active. This was further supported by the significantly lower oxygen consumption in preterm cells after treatment with oligomycin and after FCCP. The most remarkable difference was detected under FCCP treatment, which indicated that the maximal ability of the mitochondrial respiratory chain to work under increased energy demand to accelerate OCR in response to mitochondrial challenge (uncoupling) was markedly decreased in preterm cells compared to term cells. Interestingly, the cellular respiratory control ratio (RCR), the metrics of mitochondrial coupling calculated as the ratio of uncoupled to oligomycin-inhibited respiration, did not differ between groups (Fig. 4a). This indicated that in the absence of an environmental stressor or injury, mitochondria were similarly well-coupled. MitoTracker Green FM staining showed no evident abnormalities in mitochondria morphology, including loss of cristae or swelling in preterm cultures. Comparison of fluorescence signal intensity and cellular distribution showed no differences in mitochondrial mass between preterm and term cultures (Fig. 4b).

The significantly poorer response to FCCP in premature NP cells suggests decreased ability of the mitochondrial respiratory chain to meet metabolic challenge compared to term cells. Notably, Pearson’s correlation analysis showed that maximal OCR correlated negatively with the expression of selective mitochondria-associated genes encoding complex I of the electron transport chain, such as NDUFA3 and NDUFA6 but not with other complex I genes, such as NDUFV1-3 (Supplementary Fig. S2). These components have different functions; for example, NDUFA3 is required for assembly and stability of the complex I in human mitochondria, while NDUFV1 has been implicated in the response to oxidative stress^36,37^. Also, OCR showed significant negative correlation with mitochondria quality control genes, such as PINK1 (p = 0.013; R: 0.82). These observations suggest that the overall decreased mitochondrial activity and response to FCCP in preterm cells may be at least in part linked to an effect of prematurity on specific genes affecting mitochondrial integrity and function.

### Prematurity does not prevent NP progenitor cell differentiation but maintains altered gene expression and epithelial function

Our initial characterization of the cellular behavior of cultured NP progenitors performed in cells from term newborns showed that they self-renew and undergo mucociliary differentiation under ALI conditions (Fig. 1). Nevertheless, it was unclear whether preterm NP progenitors had similar capacity to differentiate under the same conditions, given their altered gene signatures and bioenergetics behavior.

**Figure 5:**
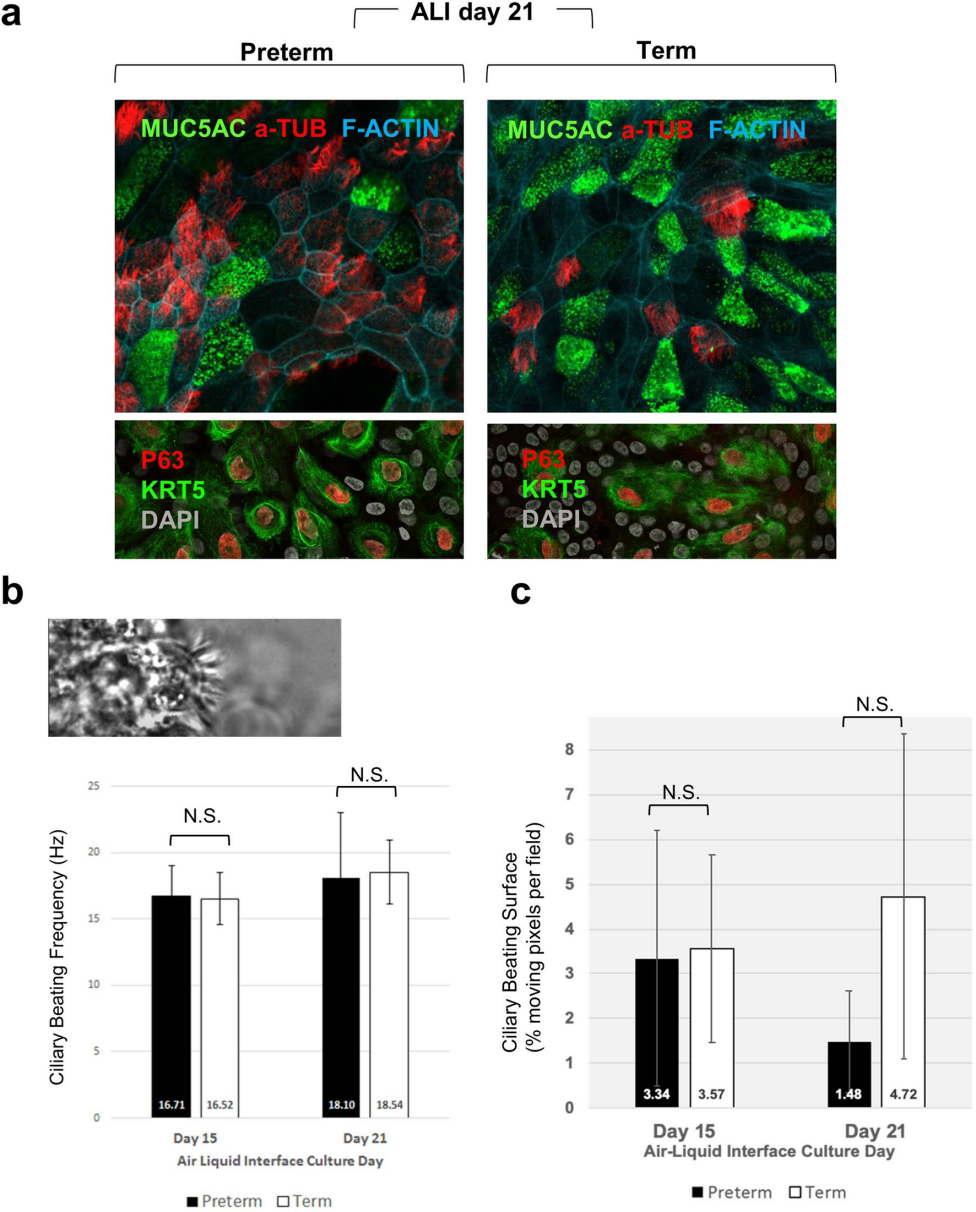
Prematurity does not prevent NP progenitor cell differentiation but compromises ciliary cell function. (**a**) Immunofluorescence of ALI day 21 organotypic cultures showing secretory (MUC5AC), multiciliated cells (acetylated a-tubulin) and basal cells (P63, KRT5) in both preterm and term epithelia (F-Actin and DAPI depicting cell boundaries and nuclei, respectively). (**b**,**c**) Functional analysis of cilia dynamics: representative picture in (**b**) of a multiciliated cell captured in the live image preparation for measurements of Ciliary Beat Frequency (CBF) and Ciliary Beating Surface (CBS) on ALI Day 15 and Day 21 organotypic cultures from preterm (n = 5) and term (n = 4). The average CBF is maintained at day 15 and day 21 in both preterm and term cultures. (**c**) The CBS is highly variable in both groups and the differences are non-significant (NS, Student *t* test). However, CBS in premature cultures tend to decline at a later stage when compared to term cultures. Graphs: bars are mean (± SED) of 10 fields/well.

To examine this issue, we performed similar experiments in preterm and term cells, allowing them to differentiate up to 21 days in ALI. Immunostaining and confocal microscopy for various markers of differentiation identified abundant multiciliated and secretory (club, goblet) cells as well as smaller population of basal cells that remained undifferentiated in a pattern of distribution indistinguishable between preterm and term cultures (Fig. 5a). We performed bulk RNAseq of ALI day 21 NP cultures to investigate whether preterm and term cells had differences in gene expression signatures that could reveal an altered balance of cell types or maturation not detected in our morphological analysis. Samples were processed and analyzed as before. Transcriptome analysis showed no significant difference in expression of gene markers of multiciliated, secretory or basal cells between preterm and term cultures, confirming our findings of preserved balance of differentiation (Supplementary Fig. S3a). To further support this, we determined the cell type abundance of our day 21 ALI cultures from their bulk RNAseq transcriptome using CIBERSORTx, a digital cytometry approach^38^. A recently published human lung tissue single-cell RNA-sequencing database^39^ was used as the signature matrix. The CIBERSORTx analysis showed no significant difference in abundance of the identified cell types between preterm and term cultures (Supplementary Fig. S3b).

We reasoned that the prematurity-associated decrease in mitochondria activity in NP progenitors, although not preventing differentiation, could have a negative impact in the function of specific cell types derived from preterm cells. Multiciliated cells require high energy for ciliary beating and cilia maintenance. Moreover, altered mitochondria function with decreased ATP production and impaired mitophagy have been associated with impaired ciliary function in cigarette smoke exposure of the airway epithelium^33,40^. Thus, we investigated whether multiciliated cells arising from differentiation of preterm progenitors were functionally different from those arising from term. Ciliary beating frequency (CBF) is a hallmark parameter widely used to assess cilia function and integrity of the mucociliary defense mechanism^41^. We used high speed video microscopy (Zeiss Live-Cell Imaging) to measure CBF in organotypic cultures derived from preterm and term at two time points: ALI culture day 15 (n = 5 each) and day 21 (n = 4 each).

Analysis of ten random fields per well, and 3 wells per donor culture, showed an average CBF between 16–18 Hz in both preterm and term NP cultures either at ALI day 15 or day 21. No significant difference in CBF was found between preterm and term cells at both stages (Fig. 5b). CBF assessed in unrelated ALI organotypic cultures from human adult tracheobronchial progenitors under the same conditions averaged ~ 6 Hz, consistent with values reported in the literature^42–44^. The lower CBF in adult cultures was interesting and could be ascribed to age-related (neonatal vs adult) or site-specific (NP vs tracheobronchial) differences in ciliary activity^45^.

We also calculated the average surface of multiciliated cells actively beating in a given field (ciliary beating surface, CBS). For this, ciliary movement was recorded by high-speed video microscopy in 10 random fields/well in organotypic cultures at ALI day 15 and then day 21. Data were analyzed using a customized script based on a Fast Fourier Transform algorithm (Matlab, Mathworks, see Methods) and expressed as percentage of moving (“beating”) pixels per field. The analysis revealed that, although with great variability, the ciliary beating surface tended to increase from ALI day 15 to 21 in term cultures but not in preterm (Supplementary Fig. S4; Fig. 5c). Instead, a trend toward a decrease in CBS of ALI day 21 preterm compared to term (31%, p = 0.097, statistically non-significant) suggested that multiciliated cells generated from preterm progenitors may not be able to sustain efficient ciliary beating at the same extent of those derived from term progenitors. Whether this trend reflected the differences in mitochondria activity of preterm progenitors and represented later effects of their program of differentiation we could only speculate.

**Figure 6:**
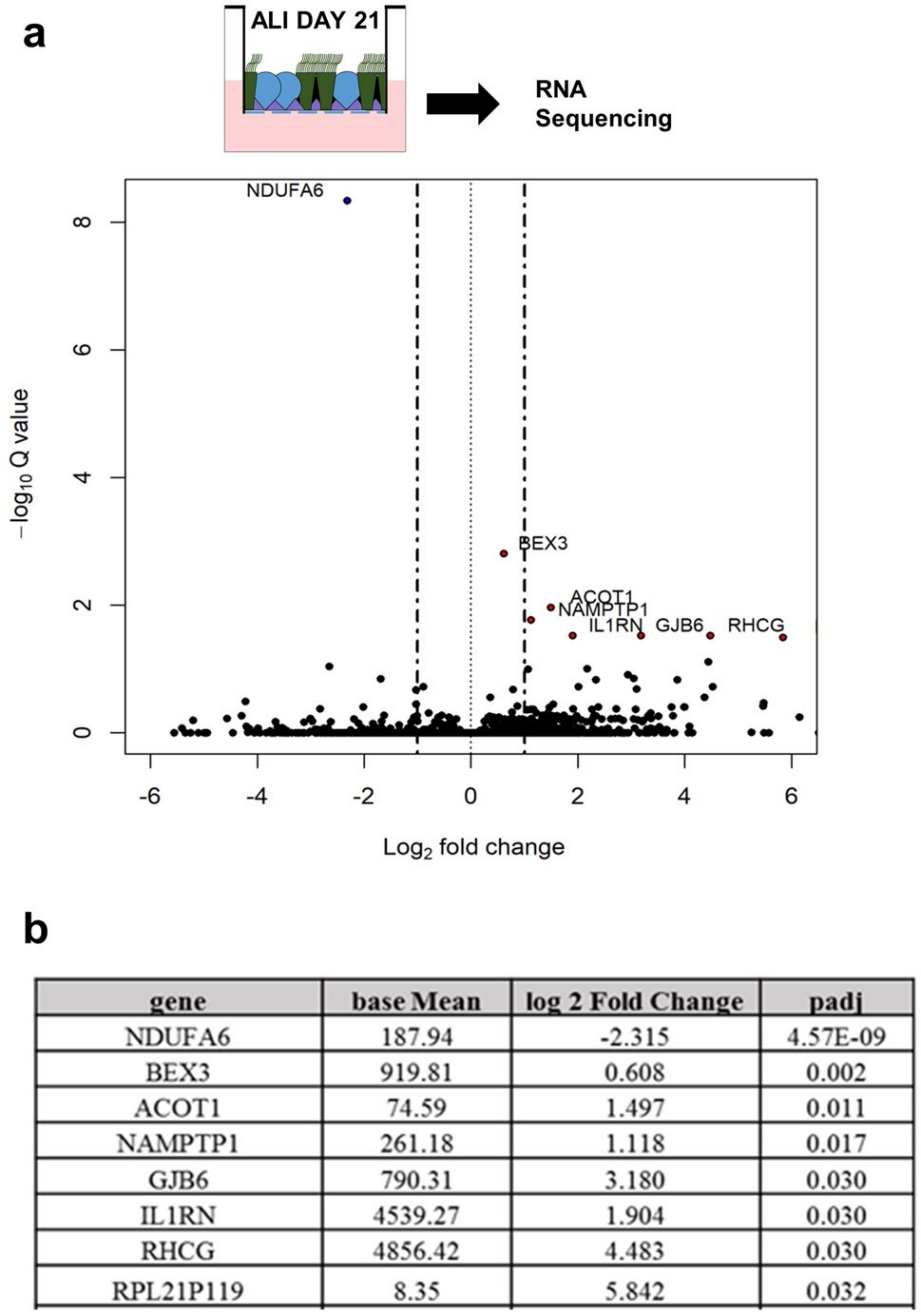
Differentiated preterm organotypic cultures have minimal but significant differences in gene expression signature compared to term cultures. Bulk RNAseq at ALI day 21 (**a**) Volcano plot showing the fold change and significance of gene expression between preterm vs term. Significantly regulated genes above and below twofold are represented. (**b**) Table listing all the differentially expressed genes between preterm vs term cultures at ALI day 21 at p-adjusted value < 0.05.

We did a survey in our transcriptome analysis of preterm and term ALI day 21 cultures searching for differentially expressed genes that could be associated with the observed difference in behavior. In contrast to our findings in the undifferentiated progenitors, a comparison of the signatures at ALI day 21 revealed only a small number of genes (8) differentially expressed between preterm and term cultures. NDUFA6 (NADH:ubiquinone oxidoreductase subunit A6) encodes a component of the mitochondria complex I oxidative phosphorylation chain whose mutations are associated with mitochondrial disease^36^ (Fig. 6, Supplementary Fig. S2b). NDUFA6 was the sole gene significantly downregulated in NP cells both prior to (ALI day 0; p-adj = 2.34 × 10^–5^; 3.4-fold) and after (ALI day 21; p-adj = 4.57 × 10^–9^; fivefold) differentiation. NDUFA6 has been implicated in viral-induced apoptosis in several cell lines. In HIV-infected cells NDUFA6 gene silencing selectively induces apoptosis, an effect that is rescued by NDUFA6 overexpression^46^. Intriguingly, this gene is upregulated in human subjects exposed to air pollution, which is thought to represent a compensatory response for the mitochondrial damage induced by environmental agents^47^. How the decreased NDUFA6 expression contributes to differences in mitochondria activity between preterm and term and inability to ramp up expression later during differentiation is currently unclear. Our survey also showed that among the few genes differentially upregulated in day 21 preterm, two are associated with stress conditions. IL1RN (IL-1 receptor antagonist, IL-1Ra), a known negative regulator of IL-1 signaling facilitates infection and is associated with frequent cycles of mitochondrial damage-repair in sepsis^48^. RHCG, a Rh family C glycoprotein is a negative regulator of NFKb expressed in airway secretory cells and upregulated in a model of squamous metaplasia in adult human bronchial epithelial cultures^49^.

Although we could not directly implicate the genes above in the preterm phenotype, they point to prematurity having a potential impact in the mechanisms that maintain energy homeostasis and integrity of the NP epithelium.

## Discussion

Here we established a minimally invasive methodology for systematic isolation and functional analysis of the stem cell behavior and differentiation program of human basal cells from nasopharyngeal aspirates. We show that this approach can be used routinely in NICU settings as an invaluable platform to investigate the impact of prematurity in the stem cell pool and barrier function of the upper respiratory tract epithelium. Due to its minimally invasive nature, it can be performed on healthy term neonates as well as extremely premature neonates, and is not limited to intubated neonates^50^, who tend to be the sickest patients. In our experience, parents are agreeable to have their neonate participate in studies on clinically discarded material. This opportunistic source of proximal airway basal cells from NP aspirates is advantageous compared to nasal brushings^51–53^, particularly in the extremely premature and clinically labile neonates.

To our knowledge, this is the first report studying the program of basal cells collected from preterm and term neonates within the initial minutes of life. The uniqueness of this approach is that isolated cells have minimal exposure to the postnatal environment before culture. NP aspirates at birth reflect the innate cell population prior to prolonged exposure of supplemental oxygen, barotrauma, dynamic microbiome, infection, or medications, all of which have the potential to change the cells’ characteristics. This allows comparing the cellular behavior and the developmental program of progenitors from distinct gestational ages. The platform is versatile and allows assessment of a broad range of parameters from gene expression signatures and morphological features to functional parameters, such as bioenergetics and ciliary dynamics.

Cell-intrinsic differences between airway epithelial cultures derived from children and adults have been reported. Basal cells isolated from pediatric tracheobronchial biopsies were shown to have higher colony-forming ability and outcompeted the adult cells in proliferation assays^54^. Here we found that premature NP progenitors carry a program of gene expression that favors cell expansion while inhibiting functions, such as mitochondria quality control and autophagic cell survival, distinctive from their term counterpart.

The activity of pulmonary mitochondrial respiratory chain complexes is developmentally regulated and in mice, increases during postnatal lung development^55^. This is consistent with our findings of differentially expressed mitochondrial complex I genes and decreased maximal respiratory capacity in preterm cells compared to term. Complex I is a crucial component of the electron transport chain and is considered the rate-limiting step of mitochondria respiration in the control of energy production. Notably, complex I dysfunction is the most common cause of mitochondrial disease in infancy and childhood and has been also implicated in age-related diseases^36^. In our study we cannot precisely identify the nature of the primary defect, but the strong correlation of decreased maximal OCR with the downregulation of PINK1 in preterm cells suggests that mechanisms that regulate mitochondria function and cellular integrity are less active. The key role of PINK1 in mitochondria quality control is widely recognized across species from *Drosophila* to mammals^34,56–58^.

Based on these observations, we speculate that preterm progenitors may be less fit to withstand continued physiologic demands over time and may have an overall maladaptive cellular response to stress compared to term cells. Indeed, these cells were unable to ramp up oxygen consumption under FCCP challenge in our assays. Moreover, the organotypic cultures differentiated from preterm NP progenitors could still maintain the ciliary beating surface area comparable to term cultures at an early stage, but not at a later stage. The accompanying differences in expression in only a small number of genes, including NDUFA6 suggest that, although not necessarily abnormal, the preterm-derived epithelium may be less fit to withstand continued or additional demands under challenging conditions. Analysis of cellular bioenergetics in the NP-derived differentiated epithelium and studying the effect of environmental exposures will provide valuable insight^59^.

We propose that NP-derived organotypic cultures has the potential to elucidate mechanisms controlling epithelial homeostasis and injury-repair of the upper respiratory tract and help understanding human neonatal diseases.

## Materials and methods

### Patient demographics and nasopharyngeal (NP) aspirate sample collection

All subjects were born at Columbia University Irving Medical Center (CUIMC) between April and August 2018. A total of 24 neonates were enrolled under this protocol, from which 11 were used in this study, after conditions for sample collection and NP cell culture were optimized. Study inclusion criteria were preterm neonates 23 0/7–30 6/7 weeks gestational age (n = 16) or healthy term neonates 37 0/7–40 6/7 weeks gestational age (n = 8), with maternal prenatal labs of rubella immune and negative hepatitis B surface antigen, Human Immunodeficiency Virus, Rapid Plasma Reagin, gonorrhea, and chlamydia tests. Exclusion criteria were neonates with suspected prenatal genetic anomaly. Neonates who clinically required suctioning to clear their upper respiratory tract airspaces in the delivery room within the first 15 min of life were considered for this study. Term infants were born via caesarian section and were admitted to the well-baby nursery. Preterm infants did not require intubation at delivery, and all received nasal bubble continuous positive airway pressure for maximum respiratory support. None were born in the setting of clinical chorioamnionitis. Prenatal care reflects contemporary medicine practices, including mothers of preterm neonates receiving at least 1 course of antenatal betamethasone for lung maturity, as clinically able for threatened preterm birth. Although oligohydramnios was detected by prenatal ultrasound in three of the preterm neonates, the respiratory support required during the NICU hospitalization was not clinically concerning for hypoplastic lungs^60^. Demographics of the NP aspirate donors used for the present study are listed in Supplementary Table S1. Appropriately sized suction catheters, selected based on the infant’s weight, were used by the neonatal delivery room resuscitation team to suction the nasopharynx and oropharynx luminal content. Aspirates (approximately 1 ml) were collected in a mucus trap. If the aspirate volume was insufficient to reach the mucus trap, the suction catheter was rinsed with 3 ml of sterile normal saline. All procedures and experiments were performed in accordance with the relevant guidelines, regulations, and protocol approved by the CUIMC institutional review board #AAAR7955. If the neonate qualified for the study, informed consent was obtained from families/parents, and the sample was coded, transferred to the laboratory on ice and stored at 4 °C.

### Expansion and differentiation of nasopharyngeal epithelial progenitors in culture

The NP aspirates were resuspended in 5 mL sterile normal saline to dilute mucus, centrifuged (4 °C, 400 rpm, 4 min) and the supernatant (including mucus) was discarded. The pellet was resuspended in 2 mL Bronchial Epithelium Growth Media (BEGM) containing penicillin–streptomycin, gentamicin, tobramycin, ceftazidime, vancomycin, and amphotericin B. Cells were seeded on 24-well collagen-coated plates (13.2 μL/mL PBS) and cultured under submerged conditions in BEGM at 37 °C, 5% CO_2_, as previously reported for HBE or tracheal epithelial cultures^14^. Media was changed 24 h after and subsequently every 48 h until cells reached approximately ~ 90% confluence (7–10 days). Cells were expanded to passage 2 prior to seeding 3.5 × 10^4^ cells on 24 well collagen-coated Transwell plates and cultured under submerged conditions in BEGM at 37 °C, 5% CO_2_. Once cells reached 100% confluence (approximately 5–7 days), the upper chamber medium was aspirated to expose cells to air–liquid-interface (ALI) and the medium in the lower chamber was replaced with Human Air Liquid Interface (hALI) media^14^ and allowed to differentiate for up to 21 days, with media changed every 48 h. Cultures were harvested for analysis at ALI day 0 (confluent, undifferentiated cells prior to ALI culture) or at days 15 and 21. In most experiments NP cultures were performed side-by side with ALI cultures of adult human tracheal progenitors (basal cells) under the same culture conditions for comparisons. These were isolated from healthy adult lung organ donors not used for transplant. Patient demographics were anonymous.

### Immunofluorescence assays

Indirect immunofluorescence was performed in NP confluent (undifferentiated progenitors) cultures and differentiated ALI organotypic cultures as described in Mori et al.^61^. Briefly, Transwell membranes were fixed with 4% (PFA) for 10 min, room temperature. For the immunofluorescence assays, membranes were washed with phosphate buffered saline (PBS) then permeabilized by 0.2% TritonX-100 in PBS for 10 min twice and blocked in 1% bovine serum albumin (BSA) for 1.5 h prior to incubation with primary and secondary antibodies. Membranes were incubated with primary antibodies in 1% BSA overnight at 4 °C: Mouse monoclonal P63 (D-9) antibody (1:50; sc-25268; Santa Cruz Biotechnology), rabbit monoclonal P63-alpha (D2K8X) XP antibody (1:200; Cell Signaling #13109), rabbit polyclonal keratin-5 antibody (1:500; # PRB-160P; BioLegend), chicken polyclonal keratin-5 antibody (1:200; #905901, BioLegend), rabbit monoclonal Nkx2.1 antibody (1:50; #76013; Abcam), rabbit polyclonal cleaved caspase-3 (Asp175) antibody (1:100; cat#9661S; Cell Signaling), mouse monoclonal Ki67 antibody (1:300; cat#550609; BD Biosciences), rabbit monoclonal acetyl-alpha-tubulin antibody (1:800; cat#5335S; Cell Signaling), mouse monoclonal mucin 5AC antibody (1:100; cat#ab3649; Abcam). F-actin was stained by Alexa Fluor™ 647 Phalloidin (1:300; A22287; Thermo Fisher). Membranes were washed with PBS prior to incubation with the appropriate secondary antibodies and 2,4-diamindino-2-phenylindole (DAPI) selected from the following and added at the corresponding dilutions and incubated in 1% BAS for 1 h at room temperature: Donkey anti-chicken IgY (IgG) (H + L), Alexa Fluor 488 nm (1:300; cat#703-545-1550; Jackson ImmunoResearch), Donkey anti-mouse IgG (H + L) highly cross-adsorbed secondary antibody, Alexa Fluor 488 nm (1:300; cat#A-21202; Thermo Fisher), Donkey anti-rabbit IgG (H + L) highly cross-adsorbed secondary antibody, Alexa Fluor 488 nm (1:300; cat#A-21206; Thermo Fisher), Donkey anti-mouse IgG (H + L) highly cross-adsorbed secondary antibody, Alexa Fluor 568 (1:300; cat#A-10037; Thermo Fisher), Donkey anti-rabbit IgG (H + L) highly cross-adsorbed secondary antibody, Alexa Fluor 568 (1:300; catalog no. A-10042; Thermo Fisher), Donkey anti-mouse IgG (H + L) highly cross-adsorbed secondary antibody, Alexa Fluor 647 (1:300; cat#A-31571; Thermo Fisher), Donkey anti-rabbit IgG (H + L) highly cross-adsorbed secondary antibody, Alexa Fluor 647 (1:300; cat#A-31573; Thermo Fisher), DAPI NucBlue™ Live ReadyProbes™ Reagent (1:10; cat#R37605; Thermo Fisher). Afterwards membranes were washed, mounted on microscope slides with ProLong Gold Antifade Mountant (cat#P10144; Thermo Fisher). Membranes were then imaged using a Zeiss confocal 710 microscope.

### Bulk RNA sequencing and analysis

Transcriptome analysis was performed in NP progenitor cultures from four term and four preterm donors selected based on their similarity in gestational age, to minimize the effect of potential differences in lung development (Supplementary Table S1). Cultures from each donor were analyzed at confluency and after differentiating for 21 days in ALI. Total RNA was extracted from NP confluent basal cells and ALI day 21 cultures with RNeasy Mini kit (#74104, Qiagen). RNA-seq libraries from Preterm 1, 2, 3, 4 and Term 1, 2, 3, 4 NP basal cells were prepared with the TruSeq Stranded mRNA kit (Illumina) following the manufacturer’s instructions. Quality of the libraries was validated by quantitation with Qubit, size analysis on a Tapestation 2200. Bar-coded cDNA libraries were pooled and sequenced on a NovaSeq 6000 (Illumina) at the JP Sulzberger Columbia Genome Center (New York, NY). Raw reads were pre-processed using FastQC^62^, After removing the adapter and low quality reads, the clean reads were mapped to the human genomes (HG38) using the TopHat (v 2.0.13). The read count was calculated using HTSeq (v.0.6). All the analysis was performed in R-3.6.3. Differential expression and principal component analysis were performed by DESeq2. Differentially expressed gene transcripts were selected based on an adjusted p-value (p-adjusted) less than 0.05. Heatmap analyses were performed using ComplexHeatmap. The heatmap depicted in Fig. 2b was generated with normalized differentially expressed genes at p < 0.05 except for KRT13, ICAM1, ITGA6, KRT14 in preterm samples undifferentiated vs ALI Day 21, and FAS, ICAM1 and IGF2R in term samples undifferentiated vs ALI Day 21. Gene Ontology testing was performed using *goseq*. For pathway analysis, a list of all genes detected in the samples was ranked on the t-statistic generated by DESeq2. This list was analyzed with the GSEA package in R using the complete Molecular Signatures Database MSigDB v7.1 gene set. Volcano plot was labeled using MASS and calibrate. CIBERSORTx^38^ was used to estimate the cell type abundances from our bulk ALI day 21 transcriptome with published human lung tissue single-cell RNA-sequencing data^39^ as signature matrix. Categories in differentiating ALI cultures included ciliated (multiciliated), secretory goblet and club cells, differentiating basal cells, and others (undetermined)^39^.

### Assessment of ciliary dynamics in NP-derived organotypic cultures

Ciliary beat frequency (CBF) was measured by high speed video microscopy and analyzed using methodologies reported in Oltean et al.^43^. Videos were captured with a Zeiss Inverted imaging system Observer.z1 equipped with Zeiss microscopy system Apotome.2 and high-speed video camera Axiocam 503. NP-derived organotypic cultures from Preterm 2, 3, 5, 6, 7 and Term 1, 2, 3, 4 subjects were imaged at ALI day 15 and day 21. Cultures were allowed to equilibrate in imaging system’s chamber at 37 °C, 5% CO_2_ for 10 min before video captured. Ten random fields per well were video captured and each culture has triplicates. Videos were recorded at 360 frames per second, typically capturing 720 total frames, using CoreView (v2.2.0.9) software. Ciliary beating frequency (CBF) was analyzed across each video using the fast Fourier transform (FFT) within Matlab (R2018a, Mathworks, Natick, MA) as previously described^43,44^. Only pixels (termed beating pixels) with magnitude greater than 1, as well as CBF between 2 and 60 Hz were included in our analysis. The percentage of ciliary beating surface (CBS) in each field was calculated by dividing the number of moving pixels by the total number of pixels in that video frame (786,432). Statistical analysis was performed using Two-tailed Student’s *t* test (Graphpad or Excel).

### Analysis of the bioenergetic function of NP-derived basal cells

Cellular mitochondrial respiration was assessed on NP-derived basal cells from all newborns listed in Supplementary Table S1 (passage 2–3) using Seahorse XFe24 Extracellular Flux Analyzer (Agilent, Santa Clara, CA) as previously reported^55,63^. Cells were seeded in collagen-coated XF 24-well cell culture microplates (Agilent) at a density of 4 × 10^4^ cells/well in 100 µL of growth medium and incubated for 2 h at 37 °C in 5% CO_2_ to attach and then another 200 µL of growth medium were added. On the next experimental day, the growth medium was replaced with 500 µL of Agilent Seahorse XF Base Medium (Agilent, 103334-100) supplemented with 2 mM glutamine (Gibco, 25030-081), 1 mM pyruvate (Gibco, 11360-070), and 10 mM glucose (Sigma, G8769). Cells were incubated for one hour prior to the assay in a non-CO_2_ incubator. Oxygen consumption rate (OCR) and extracellular acidification rate were recorded at baseline followed by the additions of 0.5 µg/mL oligomycin, 1.2 µM FCCP, and 0.5 µM rotenone plus 1 mM antimycin A. Non-mitochondrial oxygen consumption was subtracted from all OCR values, and technical replicates identified as outliers were omitted from further calculations. Values were normalized by protein content in the well. For NP-derived basal cells from each newborn donor (Supplementary Table S1), we ran at least three independent cell respiration experiments, with 5–6 technical replicates per plate, and the resulting average rate was used for the statistical analysis. Mitochondrial mass was analyzed by confocal microscopy using 50 nM MitoTracker Green FM (Invitrogen, M7514) and 2.5 µg/mL Hoechst 3342 (Invitrogen, H3570) in HBSS (Gibco, 14025). Cells were seeded in a collagen-coated 35 mm dish with 14 mm glass coverslip No.1.5 (MatTek, P35G-1.5-14-C) at a density 4 × 10^4^ cells/coverslip in the growth media. On the next experimental day, the growth media was replaced with the staining media and incubated for 20 min at 37 °C.

## Supplementary Information

Supplementary Information.

## Data Availability

All data generated or analyzed during this study are included in this published article (and Supplementary Information files) and NCBI Gene Expression Omnibus number GSE164358.
