# Supplementary material for: Prematurity alters the progenitor cell program of the upper respiratory tract of neonates
Source: Sci Rep. 2021 May 24;11:10799. doi: 10.1038/s41598-021-90093-x (PMC8144386; doi:10.1038/s41598-021-90093-x)
Supplement: Supplementary file 1 — Supplementary Information. [file 41598_2021_90093_MOESM1_ESM.pdf]

Prematurity alters the progenitor cell program of the upper respiratory tract of neonates

Jessica E. Shui[3], Wei Wang[3], Helu Liu[1], Anna Stepanova[2], Grace Liao[1,2], Jun Qian[1], Xingbin Ai[3], Vadim Ten[2], Jining Lu[4], Wellington V. Cardoso[1] *

Affiliation

[1]Columbia Center for Human Development, Columbia University Irving Medical Center, NY
[2]Division of Neonatology, Department of Pediatrics, Columbia University Irving Medical Center, NY
[3]Division of Neonatology and Newborn Medicine, Department of Pediatrics, Massachusetts General Hospital, Harvard Medical School, MA.
[4] Division of Lung Diseases, NHLBI, NIH, Bethesda, MD

A

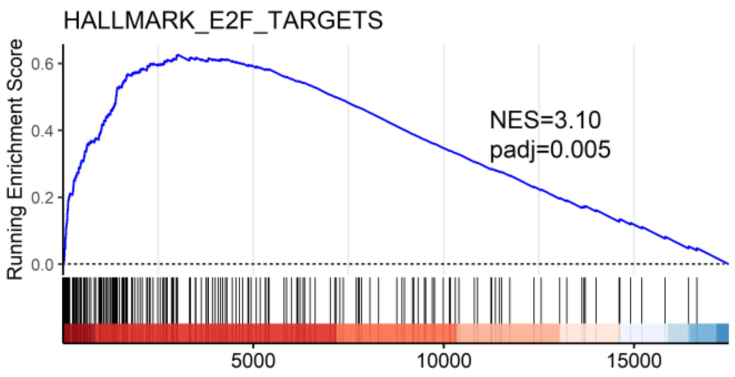

Genes	base Mean	log 2 Fold Change	p adjusted
TIMELESS	210.70	1.432	0.0000621
MCM3	553.00	1.426	0.00016034
CSE1L	1392.45	0.639	0.00019823
CDK4	959.92	0.519	0.00026553
MSH2	322.33	1.009	0.00043608
HELLS	97.51	1.814	0.00043641
GINS4	48.16	2.133	0.00045682
RAD51C	160.98	1.002	0.00054649
MCM2	314.56	1.284	0.00060019
NASP	811.93	0.722	0.00062003
MCM7	591.95	1.030	0.00079632
CDC25A	57.38	1.520	0.00094532
GINS1	61.19	1.404	0.0010104
NOP56	1116.42	1.354	0.00103675
TP53	583.98	0.795	0.00109181
RANBP1	861.77	0.794	0.00124692
XRCC6	4230.27	0.375	0.00136987
PCNA	712.51	1.392	0.00138971
MLH1	454.45	0.622	0.0015573
NUP107	331.81	0.877	0.00189584

B

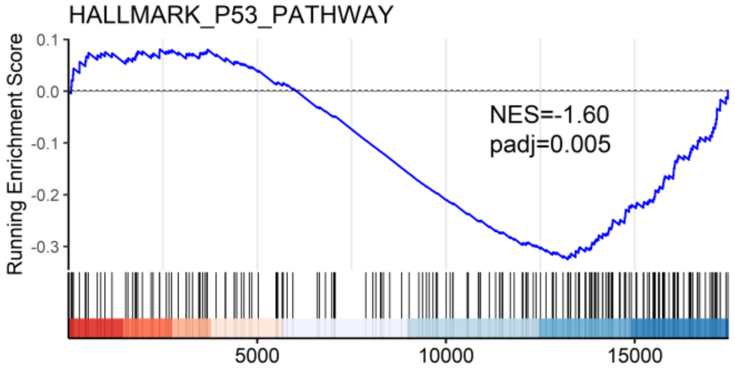

Genes	base Mean	log 2 Fold Change	p adjusted
ZNF365	64.62	-2.308	0.000056
RAD51C	160.98	1.002	0.000546
CDK5R1	132.37	-1.096	0.000648
IFI30	379.99	0.989	0.000740
TP53	583.98	0.795	0.001092
PCNA	712.51	1.392	0.001390
KIF13B	1245.47	-0.964	0.004683
PERP	14345.53	-0.609	0.004841
EI24	2208.08	0.495	0.005101
TSPYL2	238.30	0.715	0.005119
VAMP8	3458.56	-0.526	0.006657
KLF4	1210.69	-0.595	0.006780
FOXO3	1337.41	-0.782	0.007340
MXD1	1997.24	-1.234	0.008017
RGS16	25.21	1.422	0.008166
NUDT15	386.86	0.514	0.008716
RNF19B	825.97	-0.895	0.009916
CASP1	282.12	0.907	0.010162
ZFP36L1	5266.84	-0.988	0.011367
RRAD	1569.46	-1.489	0.015925

C

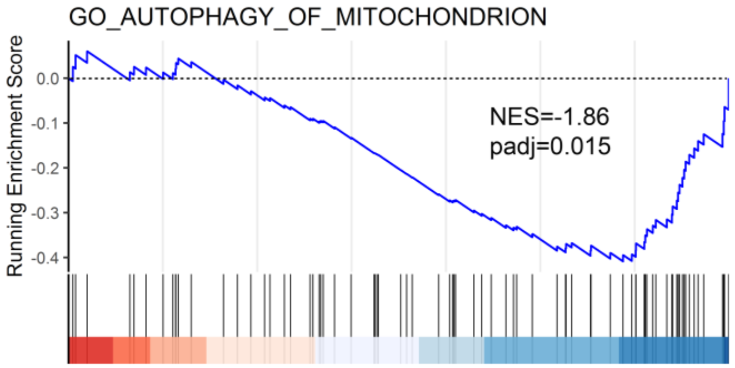

Genes	baseMean	log 2 Fold Change	p adjusted
PINK1	856.57	-0.737	8.65E-17
TP53	583.98	0.795	0.00109181
SMURF1	1096.21	-0.746	0.00131303
PRKN	25.99	-2.044	0.00195752
TIGAR	329.47	1.454	0.00223643
GABARAPL	1287.20	-0.399	0.00302045
PARK7	1852.01	0.433	0.00918916
TOMM7	1512.07	-0.414	0.0176245
MARK2	1723.82	-0.504	0.02443552
ATG9A	1155.56	-0.335	0.02926916
SREBF2	4960.71	-0.528	0.03499215
SREBF1	3285.75	-0.542	0.03927034
TSPO	2860.68	-0.555	0.04189399
GABARAP	3191.99	-0.448	0.04368527
HDAC6	538.83	-0.449	0.04771466
WIPI2	1147.22	-0.234	0.05085762
HK2	3974.13	-1.142	0.05298834
BNIP3L	2120.09	-0.679	0.05816509
USP30	284.89	-0.354	0.05929158
HIF1A	8764.61	0.397	0.0621922

Supplementary Figure S1. Gene set enrichment analysis from bulk RNAseq of undifferentiated confluent cultures of preterm and term basal cells. GSEA plots (left) depicting enrichment score for E2F targets **(A)**, P53 signaling pathway genes **(B)**, and Autophagy of mitochondria **(C)**. Top 20 representative genes. p-adjusted and fold change values are listed on corresponding tables (right).

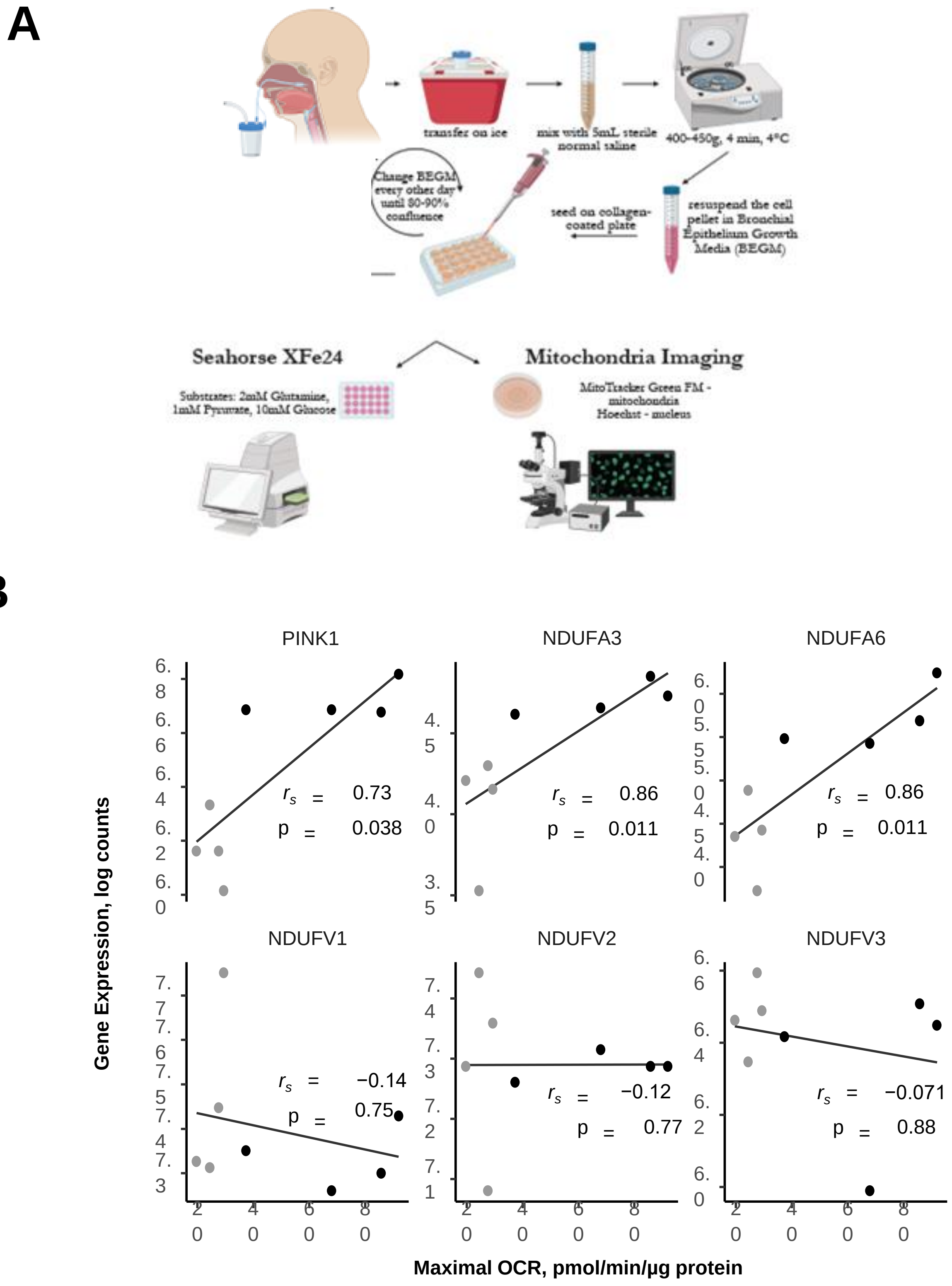

Supplementary Figure S2. **Analysis of mitochondrial respiration profile of confluent cultures of preterm and term basal cells using Seahorse XFe24 Extracellular Flux Analyzer. (A)** Schematic of the methodology, created with BioRender.com. **(B)** Correlation analysis of mitochondria-associated genes and maximal OCR in cultured NP-derived basal cells from preterm and term subjects. Reported values are Spearman's correlation coefficient (rs) and p-values; preterm infants are shown in gray.Strong correlation of OCR with mitochondria quality control (PINK1) and selected genes encoding complex I (NDUFA3, NDUFA6) but not with other genes such as NDUFV1, 2 and 3.

A

ALI day21 (preterm/term)		
**GENE MARKER**	**log2 fold change**	**p adjusted**
**SECRETORY**		
SCGB1A1	-1.509	1.0
SCGB3A2	0.246	1.0
MUC5AC	-0.357	1.0
MUC5B	0.926	1.0
SPDEF	0.489	1.0
**MULTICILIATED**		
FOXJ1	-0.134	1.0
TUBA4A	0.254	1.0
PROM1	-0.017	1.0
CDHR3	-0.394	1.0
TUBA1A	-0.219	1.0
**BASAL**		
TP63	0.423	1.0
KRT5	0.790	1.0
ITGA6	0.879	1.0
PDPN	0.822	1.0
NGFR	-0.689	1.0

B

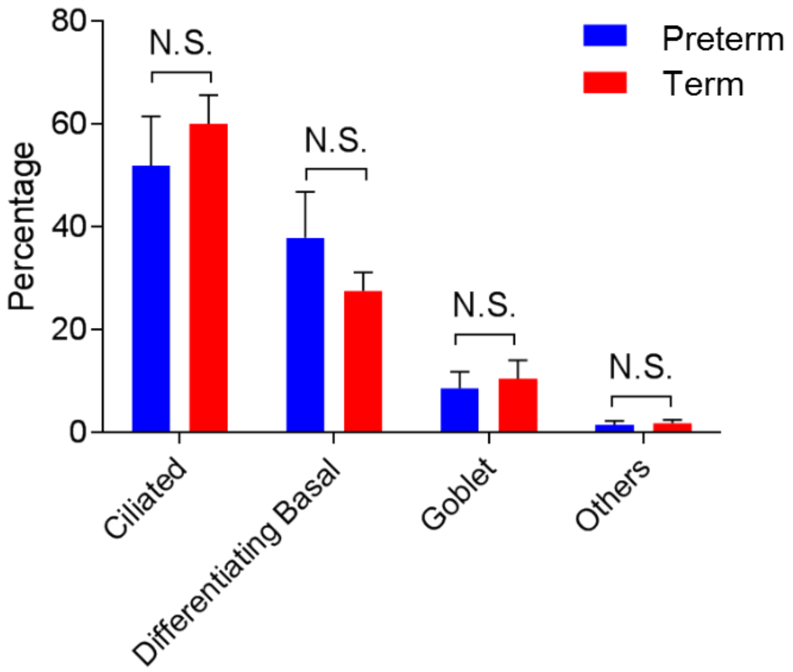

Supplementary Figure S3. Markers of airway epithelial cell differentiation from RNAseq analysis of preterm and term ALI day21 organotypic cultures. (**A**) Table showing representative markers of each cell type in ALI day 21 with respective log2fold change and p-adjusted values. No significant (NS) difference in expression of these markers between preterm and term cultures. **(B)** CIBERSORTx estimation of cell type abundance assessed from analysis of bulk RNAseq transcriptome from day 21 ALI cultures (see methods). NS difference in abundance of the airway cell types between preterm and term cultures (Student *t* test). Bars are mean (+SEM) of the percentage of cell types in each group.

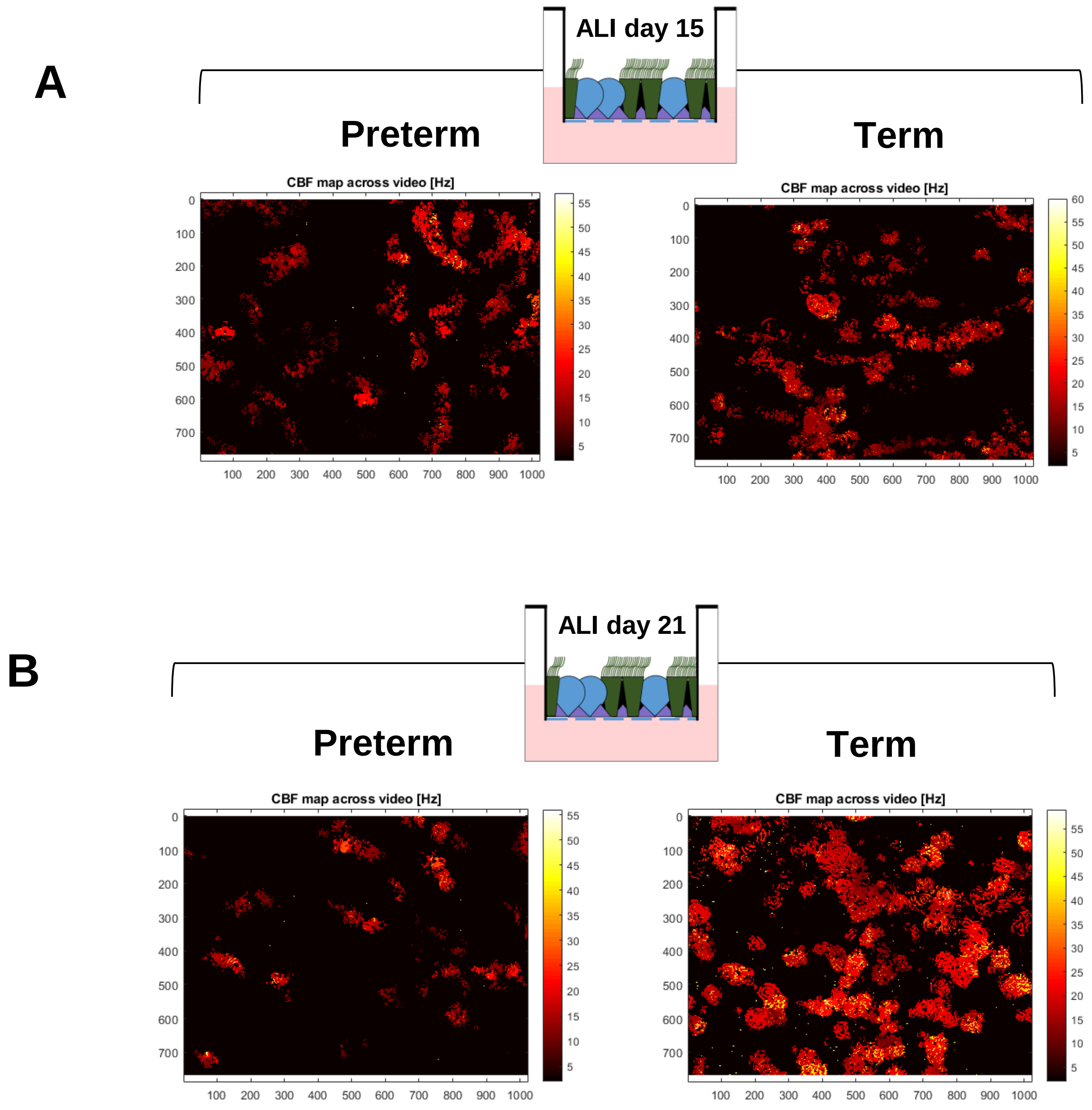

Supplementary Figure S4. Analysis of ciliary dynamics by High-Speed Video Microscopy. Representative images of ciliary beating frequency maps across a captured video (2-60Hz as indicated by the colored bar) in NP-derived ALI day 15 (a) and day 21 (b) cultures from preterm and term subjects (please also see Fig. 5 and Methods).

Study patient number	Gestational Age (weeks)	Birth Weight (grams)	Sex	Maternal Comorbidities/ Fetal Complications	Multiple Gestation	Total number of antenatal betamethasone doses[1]	Amniotic Fluid Level	Highest FiO_2 During Delivery Resuscitation (%)[2]	Positive Pressure Ventilation During Delivery Resuscitation
Preterm 1	29	1200	Female	GDMA2 on insulin[3], PPROM for 12 days[4], Placental pathology with funisitis with sterile culture[5]	No	4	Oligohydramnios[6]	21	Yes
Preterm 2	29	1110	Male	Reverse TTTS[7], preterm labor	Yes	2	Oligohydramnios	50	No
Preterm 3	28	1345	Male	Maternal hypertension[8]	No	2	Normal	30	No
Preterm 4	27	860	Male	PPROM for 10 days, IUGR[9], preterm labor	No	3	Oligohydramnios	100	Yes
Preterm 5	25	495	Female	IUGR, HELLP syndrome[10]	No	2	Normal	40	No
Preterm 6	29	980	Female	Preeclampsia with severe features[8]	No	2	Normal	40	Yes
Preterm 7	29	1310	Male	Maternal anemia, laser and amnioreduction for TTTS, preterm labor	Yes	2	Polyhydramnios[11]	50	No
Term 1	40	3795	Male	Maternal obesity[12], fetal intolerance of labor	No	0	Normal	21	No
Term 2	40	2985	Female	Arrest of labor, fetal intolerance of labor	No	0	Normal	21	No
Term 3	37	3530	Female	Prior caesarian section	No	0	Normal	21	No
Term 4	37	2890	Female	Arrest of labor	No	0	Normal	21	No

Supplementary Table S1. Perinatal clinical data of newborn donors (nasopharyngeal aspirates).
Clinical characteristics of patients and respiratory related environmental exposures at the time of NP progenitor cell collection. Definition of Abbreviations: FiO2 = fraction of inspired oxygen; GDMA2 = gestational diabetes mellitus type 2; PPROM = preterm premature rupture of membranes; TTTS = Twin to twin transfusion syndrome; IUGR = intrauterine growth restriction; HELLP syndrome = Hemolysis, elevated liver enzymes, low platelet count syndrome.

Supplemental References:

1 McGoldrick E, S. F., Parker R, Dalziel SR. Antenatal corticosteroids for accelerating fetal lung maturation for women at risk of preterm birth. *Cochrane Database Syst Rev* **12**, doi:10.1002/14651858.CD004454.pub4 (2020).

2 Kim E, N. M. Oxygen Therapy for Neonatal Resuscitation in the Delivery Room. *Neoreviews* **20(9)**, e500-e512, doi:10.1542/neo.20-9-e500 (2019).

3 Mitanchez D, Y. C., Siddeek B, Boubred F, Benahmed M, Simeoni U. The offspring of the diabetic mother--short- and long-term implications. *Best Pract Res Clin Obstet Gynaecol* **29(2)**, 256-269, doi:10.1016/j.bpobgyn.2014.08.004 (2015).

4 Tchirikov, M. *et al.* Mid-trimester preterm premature rupture of membranes (PPROM): etiology, diagnosis, classification, international recommendations of treatment options and outcome. *Journal of Perinatal Medicine* **46**, 465-488, doi:10.1515/jpm-2017-0027 (2018).

5 Kim, C. J. *et al.* Acute chorioamnionitis and funisitis: definition, pathologic features, and clinical significance. *American Journal of Obstetrics and Gynecology* **213**, S29-S52, doi:10.1016/j.ajog.2015.08.040 (2015).

6 Wu CS, C. C., Chou HC. Pulmonary Hypoplasia Induced by Oligohydramnios: Findings from Animal Models and a Population-Based Study. *Pediatr Neonatol* **58(1)**, 3-7, doi:10.1016/j.pedneo.2016.04.001 (2017).

7 Bamberg, C. & Hecher, K. Update on twin-to-twin transfusion syndrome. *Best Practice & Research Clinical Obstetrics & Gynaecology* **58**, 55-65, doi:10.1016/j.bpobgyn.2018.12.011 (2019).

8 Sutton ALM, H. L., Tita ATN. Hypertensive Disorders in Pregnancy. *Obstet Gynecol Clin North Am* **45(2)**, 333-347, doi:10.1016/j.ogc.2018.01.012 (2018).

9 forMaternal-FetalMedicin, A. C. o. O. a. G. C. o. P. B. O. a. t. S. ACOG Practice Bulletin No. 204: Fetal Growth Restriction. *Obstet Gynecol* **133(2)**, e97-e109, doi:10.1097/AOG.0000000000003070 (2019).

10 Burwick RM, F. B. Complement activation and regulation in preeclampsia and hemolysis, elevated liver enzymes, and low platelet count syndrome. *Am J Obstet Gynecol* **S0002-9378(20)**, 31129-31127, doi:10.1016/j.ajog.2020.09.038 (2020).

11 Hwang DS, B. B. in *StatPearls [Internet]* Vol. 2021 Jan (StatPearls Publishing, 2021).

12 Howell KR, P. T. Effects of maternal obesity on placental function and fetal development. *Reproduction* **153(3)**, R97-R108, doi:10.1530/REP-16-0495 (2017).
